# Risk of ischemic stroke after discharge from inpatient surgery: Does the type of surgery matter?

**DOI:** 10.1371/journal.pone.0206990

**Published:** 2018-11-05

**Authors:** Cheng-Yang Hsieh, Chin-Wei Huang, Darren Philbert Wu, Sheng-Feng Sung

**Affiliations:** 1 Department of Neurology, Tainan Sin Lau Hospital, Tainan, Taiwan; 2 School of Pharmacy, Institute of Clinical Pharmacy and Pharmaceutical Sciences, College of Medicine, National Cheng Kung University, Tainan, Taiwan; 3 Department of Neurology, National Cheng Kung University Hospital and College of Medicine, Tainan, Taiwan; 4 Division of Neurology, Department of Internal Medicine, Ditmanson Medical Foundation Chiayi Christian Hospital, Chiayi City, Taiwan; 5 Department of Information Management and Institute of Healthcare Information Management, National Chung Cheng University, Chiayi County, Taiwan; Beth Israel Deaconess Medical Center, UNITED STATES

## Abstract

**Objective:**

Stroke is a well-known and devastating complication during the perioperative period. However, detailed stroke risk profiles within 90 days in patients discharged without stroke after inpatient surgery are not fully understood. Using the case-crossover design, we aimed to evaluate the risk of ischemic stroke in these patients.

**Methods:**

We included adult patients with the first hospitalization for ischemic stroke between 2011 and 2012 from 23 million enrollees in the National Health Insurance Research Database. Admission date of the hospitalization was defined as the case day and exactly 365 days before the admission date as the control day. The exposure was the last hospitalization for surgery within 1–30, 31–60, or 61–90 days (case period) before the case day or similar time intervals (control period) before the control day. Surgical types were grouped based on the International Classification of Diseases procedure codes. We performed conditional logistic regression adjusting for time-varying variables to determine the relationship between surgery and subsequent stroke, and case-time-control analyses to examine whether the results were confounded by the time-trend in surgery.

**Results:**

A total of 56596 adult patients (41% female, mean age 69 years) comprised the study population. After adjustment was made for confounding variables, an association between stroke and prior inpatient surgery within 30 days was observed (adjusted odds ratio 1.44; 95% confidence interval 1.29–1.61). Cardiothoracic, vascular, digestive surgery, and musculoskeletal surgery within 30 days independently predicted ischemic stroke in the case-crossover analysis. In the case-time-control analysis, inpatient surgery remained an independent risk factor for ischemic stroke, whereas only cardiothoracic, vascular, and digestive surgery independently predicted ischemic stroke.

**Conclusions:**

Surgery as a whole independently increased the risk of ischemic stroke within 30 days. Among various types of surgery, cardiothoracic, vascular, and digestive surgery significantly increased the risk of ischemic stroke.

## Introduction

Stroke is among the most feared and devastating complications after surgery. Perioperative stroke, as defined by Mashour et al as “a brain infarction of ischemic or hemorrhagic etiology that occurs during surgery or within 30 days after surgery” [1], is associated with more severe neurological deficits at discharge, longer hospital stays [2], and even an eight-fold increase in the mortality risk [3] as compared to stroke without recent antecedent surgery. Its incidence varies depending on the type of surgery and ranges between 1.9–9.7% in cardiovascular surgery and 0.1–1.9% in non-cardiac, non-neurologic, and non-major vascular surgery [4]. Associated risk factors, such as age, sex, genetic factors, life styles, comorbidities, and the underlying disease leading to the need for a surgical procedure, also have an impact on the risk of perioperative stroke [2,4].

Knowing the increase in stroke risk after surgery relative to the background risk may be helpful for pre-surgical doctor-patient communication and shared decision-making. Nevertheless, most prior studies have focused on the absolute stroke incidence in the surgical population [3,5–7], rather than the relative risk compared to a matched population without surgery. Although surgery other than cardiovascular and neurological surgery is generally considered low risk for perioperative stroke [3], the risk of ischemic stroke and transient ischemic attack remains increased up to 90 days after non-carotid and non-cardiac surgery [2]. In other words, the risk of stroke is still substantial even after the perioperative period. However, detailed stroke risk profiles within 90 days in patients discharged after various types of surgery are not yet fully understood.

Because the exposure to surgery is short and its effect on the risk of stroke is short-term and declines with time [2], self-controlled observational study designs, such as the case-crossover design [8], may suit the purpose of investigating the relationship between surgery and stroke. Relatively time-invariant confounders, such as genetic disposition, comorbidities, or life styles, can be minimized with the case-crossover study design [9,10]. Using the case-crossover design, we aimed to evaluate the association between stroke and different types of inpatient surgery within 90 days before the stroke in a nationwide population.

## Materials and methods

### Data source

The National Health Insurance of Taiwan, launched in Mar 1995, is a single-payer health care program, which provides universal access to inpatient care, outpatient care, dental care, and prescription medications for over 99.5% of its residents [11]. In this study, we used a specific dataset from the National Health Insurance Research Database (NHIRD), which consists of the claims data of all patients who were hospitalized with stroke during 2011 to 2012. Therefore, virtually all hospitalized stroke patients in Taiwan during this time period were captured in this study.

Because all information in the database has been de-identified, this study was exempt from a full review by the Institutional Review Board of Ditmanson Medical Foundation Chia-Yi Christian Hospital (CYCH-IRB No. 106101) and the need for informed consent was waived.

### Study design and population

**Fig 1:**
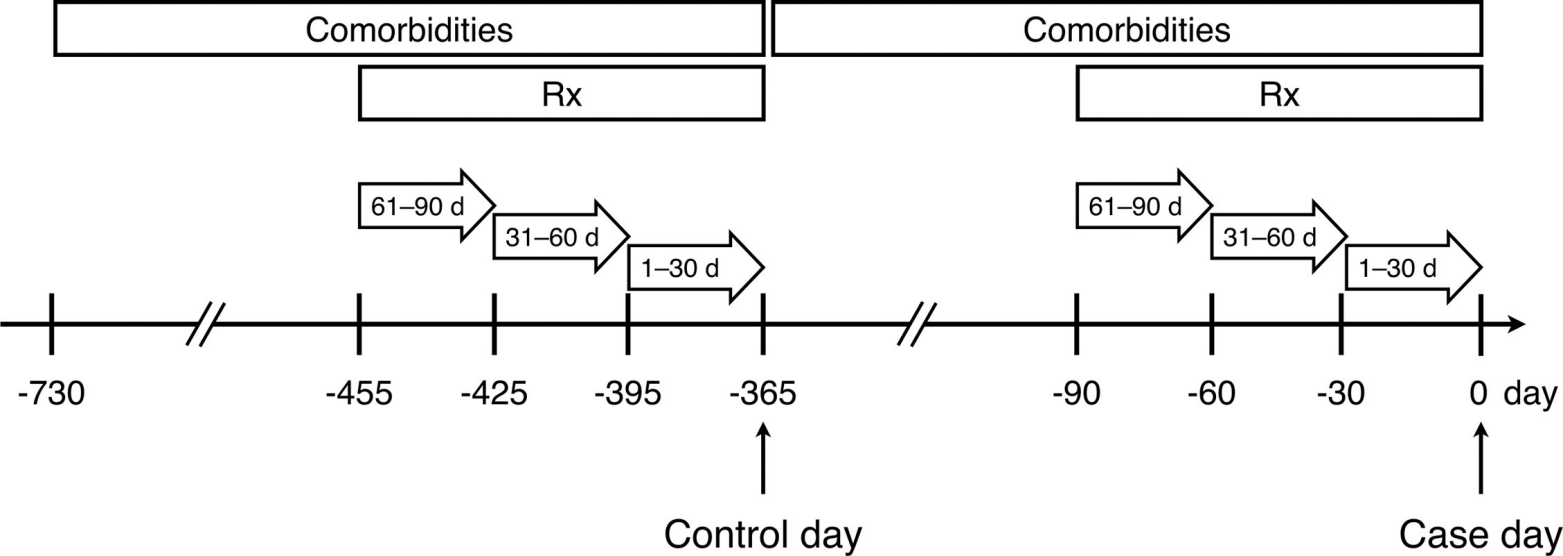
Schematic illustration of the study design. Comorbidities were ascertained using the claims within 365 days while medications were obtained from the claims within 90 days. Three risk time periods (1 to 30, 31 to 60, and 61 to 90 days) were evaluated.

This study used a case-crossover design. S1 Fig illustrates the flowchart of the study population. We selected the first hospitalization for ischemic stroke for each patient between Jan 2011 and Dec 2012 from the NHIRD based on the principal discharge diagnosis code (International Classification of Diseases, Ninth Revision, Clinical Modification [ICD-9-CM] diagnosis codes 433.xx and 434.xx) [12,13]. We defined the admission date as the case day and exactly 365 days before the admission date as the control day so as to prevent the potential seasonal effects on stroke incidence and to ensure a sufficient wash-out period (Fig 1). Patients with any type of prior stroke (ICD-9-CM diagnosis codes 430 to 434, 436, or 438) recorded in the inpatient or outpatient claims, regardless of principal or secondary diagnosis, within two years before the case day were excluded and, therefore, patients whose stroke occurred during the same hospitalization when surgery was performed were not included in this study. Patients younger than 20 years of age and those without claims items in the database were also eliminated. The remaining patients comprised the study population.

### Exposure

We evaluated three risk time periods (1 to 30, 31 to 60, and 61 to 90 days). The previous exposure was defined as the last hospitalization for surgery within 1 to 30, 31 to 60, or 61 to 90 days (case period) before the case day or similar time intervals (control period) before the control day (Fig 1). We obtained the principal ICD-9-CM procedure code and stratified cases by surgical types based on ICD-9-CM procedure codes. (S1 Table). The mode of anesthetic procedure used for the surgery was classified as general anesthesia or other anesthesia (epidural, spinal, or local).

### Variables

We retrieved baseline demographics and comorbidities including hypertension, diabetes mellitus, hyperlipidemia, atrial fibrillation (AF), coronary artery disease, congestive heart failure, chronic kidney disease, chronic obstructive pulmonary disease, peripheral artery disease, transient ischemic attack, and cancer. Comorbidities were ascertained using validated algorithms [14] based on ICD-9-CM diagnosis codes (S2 Table) from the inpatient and outpatient claims in the one-year lookback period before the case day or the control day (Fig 1). Information on health care utilization (number of outpatient visits) and medication use during 1 to 90 days before the case day or the control day were collected. Use of antidiabetic drugs, antihypertensives, lipid lowering agents, antiplatelets, oral anticoagulants, non-steroidal anti-inflammatory drugs, and antipsychotics were grouped according to the anatomical therapeutic chemical classification system (S3 Table). Non-steroidal anti-inflammatory drugs and antipsychotics were evaluated because they have been found to increase the risk of ischemic stroke [15–18].

### Statistical analysis

We used McNemar’s tests to compare the proportion of comorbidities and medication use and the Wilcoxon signed-rank test to assess differences in health care utilization between the case period and the control period. We performed conditional logistic regression to determine the relationship between surgery and subsequent stroke. Because of a relatively long time interval between the case and control periods, variables such as comorbidities, health care utilization, and use of medications should be considered to be within-subject time-varying covariates. Therefore, in addition to univariate analyses, we performed multivariate analyses in which adjustment was made for these time-varying confounding variables to account for the discordance of comorbidities, health care utilization, and use of medications between the case and control periods. Because general anesthesia has been found to increase the risk of postoperative stroke in certain types of surgery [19,20], additional analyses were performed by including the mode of anesthesia in the model. In sub-analyses for different types of surgery separately, we repeated multivariate analyses by excluding patients undergoing types of surgery other than the type of interest.

The case-crossover design has an inherent weakness that it is sensitive to the time-trend in exposure. In other words, if there is a significantly increased chance of the exposure (undergoing surgery) at the time near the endpoint (case period) than at an earlier reference point (control period), the odds ratio of the exposure will be elevated, thus creating a spurious, non-causal association [9]. To examine whether our study is confounded by such a time-trend in surgery, we performed case-time-control analyses [21]. A stroke-free cohort of adults who were alive at the end of 2012 and used health care services between Jan 2011 and Dec 2012 was assembled from a dataset of one million enrollees randomly sampled from the NHIRD. Each of the patient with ischemic stroke was matched to a randomly-selected control from the stroke-free cohort by age and sex. Controls were assigned the same case day as the matched cases. Variables were obtained in the same way as in the case-crossover study. We performed conditional logistic regression by including the interaction term between surgery and group (case with ischemic stroke versus control without ischemic stroke) [21].

Two-tailed *p* values <0.05 were considered statistically significant. Statistical analyses were performed using Stata 15.1 (StataCorp, College Station, Texas).

## Results

We identified 73062 patients hospitalized for ischemic stroke between 2011 and 2012. After excluding those who did not meet the study criteria (S1 Fig), the remaining 56596 adult patients (41% female, mean age 69 years) comprised the study population. Table 1 gives the proportions of comorbidities, health care utilization, and proportions of medication use during the case period and control period. As compared with the control period, a higher proportion of patients were diagnosed with comorbidities and were prescribed medications during the case period. In addition, a greater utilization of health care was observed during the case period. Table 2 gives the types of surgery. Digestive, cardiothoracic, and musculoskeletal surgery dominated among patients.

**Table 1:** Comorbidities and medication use before incident hospitalization for ischemic stroke.

	Case period (n = 56596)	Control period (n = 56596)	*P*
**Comorbidity, %**			
**Hypertension**	55.0	51.0	<0.001
**Diabetes mellitus**	30.6	28.8	<0.001
**Hyperlipidemia**	19.0	18.1	<0.001
**Atrial fibrillation**	5.1	4.0	<0.001
**Coronary artery disease**	16.3	15.0	<0.001
**Congestive heart failure**	9.0	7.1	<0.001
**Chronic kidney disease**	4.9	3.7	<0.001
**Chronic obstructive pulmonary disease**	9.5	8.6	<0.001
**Peripheral artery disease**	3.4	2.9	<0.001
**Transient ischemic attack**	2.2	1.7	<0.001
**Cancer**	5.4	4.4	<0.001
**Health care utilization, median (IQR)**	2 (0–4)	1 (0–3)	<0.001
**Medication, %**			
**ACE inhibitors or ARBs**	33.9	29.5	<0.001
**Beta blockers**	24.4	21.5	<0.001
**Calcium channel blockers**	33.9	31.9	<0.001
**Diuretics**	16.3	14.2	<0.001
**Other antihypertensives**	6.2	5.5	<0.001
**Oral antidiabetic drugs**	25.3	24.0	<0.001
**Insulins**	5.4	4.4	<0.001
**Lipid lowering agents**	15.5	14.3	<0.001
**Antiplatelets**	27.1	24.6	<0.001
**Oral anticoagulants**	2.1	1.8	<0.001
**NSAIDs**	38.8	35.8	<0.001
**Antipsychotics**	6.7	4.8	<0.001

ACE, angiotensin-converting enzyme; ARB, angiotensin receptor blocker; IQR, interquartile range; NSAID, nonsteroidal anti-inflammatory drug.

**Table 2:** Surgery performed during the case and control periods.

	Case period			Control period		
	1–30 d	31–60 d	61–90 d	1–30 d	31–60 d	61–90 d
**Nervous**	20 (2.0)	15 (2.0)	11 (1.7)	15 (2.6)	8 (1.4)	3 (0.6)
**Eye**	25 (2.4)	15 (2.0)	16 (2.5)	17 (2.9)	21 (3.7)	8 (1.5)
**Ear/nose/mouth/pharynx**	17 (1.7)	14 (1.8)	17 (2.6)	19 (3.3)	12 (2.1)	12 (2.3)
**Cardiothoracic**	209 (20.4)	136 (17.7)	127 (19.4)	79 (13.6)	83 (14.7)	85 (16.4)
**Vascular**	91 (8.9)	92 (12.0)	67 (10.3)	45 (7.7)	51 (9.1)	43 (8.3)
**Digestive**	295 (28.8)	226 (29.4)	174 (26.6)	140 (24.1)	149 (26.5)	154 (29.7)
**Genitourinary**	81 (7.9)	55 (7.2)	52 (8.0)	60 (10.3)	40 (7.1)	42 (8.1)
**Obstetric/gynecologic**	10 (1.0)	8 (1.0)	5 (0.8)	7 (1.2)	9 (1.6)	7 (1.4)
**Musculoskeletal**	196 (19.2)	159 (20.7)	135 (20.7)	152 (26.1)	161 (28.6)	129 (24.9)
**Integumentary**	67 (6.6)	42 (5.5)	47 (7.2)	43 (7.4)	22 (3.9)	31 (6.0)
**Miscellaneous**	12 (1.2)	6 (0.8)	2 (0.3)	5 (0.9)	7 (1.2)	4 (0.8)
**Total**	1023 (100)	768 (100)	653 (100)	582 (100)	563 (100)	518 (100)

Data are numbers (percentage).

**Fig 2:**
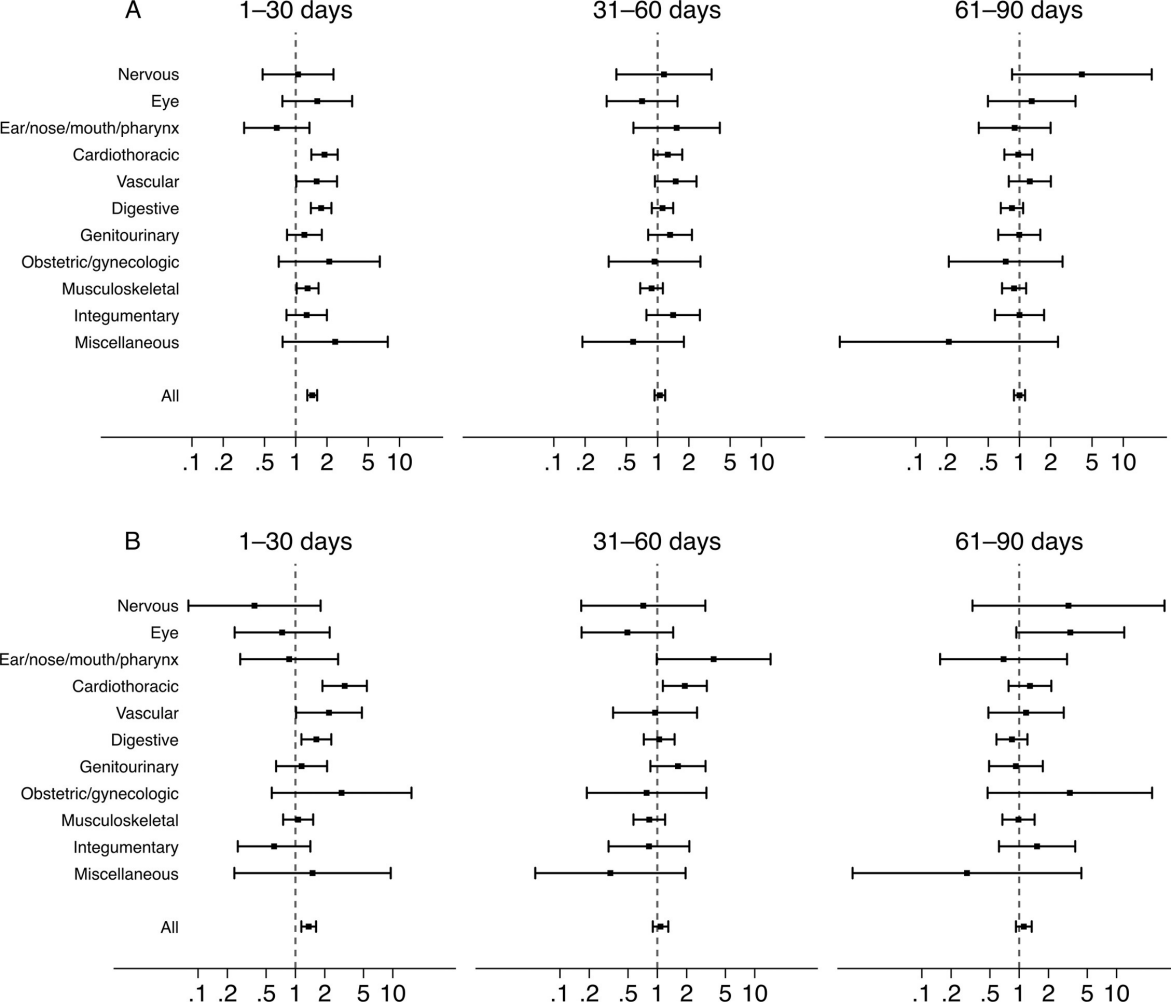
Risk of ischemic stroke across various types of surgery. Adjusted odds ratios and 95% confidence intervals for various types of surgery associated with ischemic stroke within 1–30, 31–60, and 61–90 days using case-crossover analysis (A) and case-time-control analysis (B). Adjustment was made for discordance of comorbidities, health care utilization, use of medications, and mode of anesthesia.

Table 3 lists the crude and adjusted odds ratios for surgery performed within 3 time periods. In the univariate analyses, surgery was associated with an increased risk of ischemic stroke within up to 90 days and the magnitude of the risk decreased as the time interval increased. After adjustment was made for confounding variables, an increased risk of stroke was observed only between 1 to 30 days after surgery. Fig 2A shows the adjusted odds ratios and their 95% confidence intervals for various types of surgery. Cardiothoracic, vascular, digestive surgery, and musculoskeletal surgery independently predicted ischemic stroke within 30 days, whereas other types of surgery were not associated with an increased risk of stroke (S4 Table).

**Table 3:** Risk of ischemic stroke associated with surgery (n = 56596).

Surgery	No. of patients undergoing surgery during case period	No. of patients undergoing surgery during control period	Crude OR (95% CI)	Adjusted OR^a^ (95% CI)	Adjusted OR^b^ (95% CI)
**1–30 d**	1023	582	1.79 (1.62–1.99)	1.41 (1.26–1.57)	1.44 (1.29–1.61)
**31–60 d**	768	563	1.38 (1.24–1.55)	1.03 (0.91–1.16)	1.05 (0.93–1.18)
**61–90 d**	653	518	1.27 (1.13–1.43)	0.98 (0.87–1.11)	1.00 (0.88–1.13)

OR, odds ratio; CI, confidence interval.

^a^Adjusted for discordance of comorbidities, health care utilization, and use of medications.

^b^Adjusted for discordance of comorbidities, health care utilization, use of medications, and mode of anesthesia.

In the case-time-control analysis, 2212 cases were unable to be matched with any controls. Therefore, a total of 54384 cases and 54384 controls were included in the analysis. As compared with the control group, patients in the case group were more likely to be diagnosed with comorbidities and were more likely to be prescribed medications (S5 Table). Table 4 shows the results of the case-time-control analysis. The crude and adjusted odds ratios in row A indicate the effect of natural time trends in surgery. The odds ratios in row B, which were obtained from the interaction term between surgery and group, stand for the net effect of surgery on stroke occurrence. The increased risk of stroke associated with surgery persisted in the case-time-control analysis. Fig 2B shows the adjusted odds ratios and their 95% confidence intervals for various types of surgery. Cardiothoracic surgery within 60 days independently predicted ischemic stroke, whereas vascular surgery and digestive surgery within 30 days was associated with an increased risk of ischemic stroke (S4 Table).

**Table 4:** Risk of ischemic stroke associated with surgery using case-time-control analyses (n = 54384).

	Case-crossover			Case-time-control		
Surgery	Crude OR (95% CI)	Adjusted OR^a^ (95% CI)	Adjusted OR^b^ (95% CI)	Crude OR (95% CI)	Adjusted OR^a^ (95% CI)	Adjusted OR^b^ (95% CI)
**A. 1–30 d**	1.76 (1.58–1.95)	1.38 (1.24–1.55)	1.42 (1.27–1.58)	1.17 (1.03–1.34)	1.02 (0.89–1.17)	1.04 (0.91–1.20)
**B. 1–30 d × G**^c^				1.50 (1.27–1.78)	1.38 (1.16–1.64)	1.37 (1.15–1.63)
**A. 31–60 d**	1.35 (1.20–1.51)	1.00 (0.89–1.13)	1.02 (0.91–1.15)	1.10 (0.96–1.26)	0.94 (0.82–1.08)	0.96 (0.84–1.11)
**B. 31–60 d × G**^c^				1.22 (1.02–1.46)	1.09 (0.90–1.30)	1.08 (0.90–1.30)
**A. 61–90 d**	1.28 (1.14–1.44)	0.99 (0.88–1.12)	1.01 (0.89–1.15)	1.04 (0.91–1.20)	0.91 (0.79–1.04)	0.92 (0.80–1.06)
**B. 61–90 d× G**^c^				1.23 (1.02–1.47)	1.12 (0.93–1.35)	1.11 (0.93–1.34)

OR, odds ratio; CI, confidence interval.

^a^Adjusted for discordance of comorbidities, health care utilization, and use of medications.

^b^Adjusted for discordance of comorbidities, health care utilization, use of medications, and mode of anesthesia.

^c^G = 1 for cases with stroke and G = 0 for controls.

## Discussion

We found that surgery as a whole increased the risk of ischemic stroke within 30 days, independent of comorbidities, health care utilization, medication use, and mode of anesthesia, in patients who were discharged free of stroke after surgery. The observed risk persisted in the case-time-control analysis, in which time trends in surgery was accounted for. On the other hand, the risk of ischemic stroke varied across types of surgery. Cardiothoracic, vascular, and digestive surgery consistently predicted ischemic stroke within 30 days in both the case-crossover and case-time-control analyses, whereas the remaining types of surgery were not associated with an increased risk of ischemic stroke.

Stroke generally afflicts patients with underlying vascular risk factors; however, it could be triggered by an acute event such as surgery [22]. When investigating the relationship between surgery and stroke, a conventional cohort or case-control approach may at times encounter confounding issues [9]. For example, smoking, sedentary life styles, obesity, family history of stroke, hypertension, and diabetes are well known risk factors for stroke, but also contributed to the chance of undergoing cardiac and vascular surgery, such as coronary artery bypass grafting (CABG) and carotid endarterectomy. Consequently, it may be difficult to fully account for all these confounders in a conventional study design, particularly when using secondary healthcare data for research. In contrast, relatively time-invariant confounders (e.g., life styles, family history, socioeconomic status) can be effectively controlled in a case-crossover study [9,10]. With the case-crossover design, we confirmed the association between surgery and stroke and also provided estimates about the risk of stroke in different types of surgery.

Inasmuch as stroke has manifold mechanisms, the timing of stroke after surgery may depend on the individual etiologic mechanism of stroke, which varies across types of surgery. For example, in stroke secondary to CABG, the most common mechanisms included embolism and hypoperfusion [23]. Perioperative embolism may originate from intracardiac thrombi, vulnerable atheroma in the aortic arch, or atherosclerotic lesions in large arteries because of manipulation of the above structures during surgery [24–26]. Intraoperative hypotension may cause watershed infarction in patients with pre-existing large artery steno-occlusive disease [27]. Most strokes due to these mechanisms are supposed to occur during or immediately after surgery, as shown in a study which found that nearly two thirds of strokes occurred within two days after CABG [23]. In addition to clinically evident stroke, new silent brain infarcts were detected on diffuse-weighted imaging in about a quarter of patients who underwent CABG on day 3 after the procedure [28]. Nevertheless, some other mechanisms, such as withdrawal of pre-existing antithrombotic treatment or embolism due to new-onset AF, must play a role in patients with late stroke after surgery.

New-onset AF is common in patients after cardiac surgery, with an incidence varying between 20% and 50% [29]. It is a major cause of postoperative stroke and occurred at a mean of 6 days after CABG. [30] However, postoperative AF is not exclusive to cardiac surgery [31]. For non-cardiac surgery, a study found a 9% incidence of postoperative new-onset AF among patients with more critical illness [32]. In our study, apart from cardiothoracic and vascular surgery, only digestive surgery was associated with an increased risk of stroke. This is in line with a previous finding of a 0.7% incidence of stroke after hemicolectomy in contrast to a 0.2% incidence of stroke after total hip replacement [5]. This may be partly because patients undergoing digestive surgery are more critically ill than those undergoing operations on non-vital organs and thus had a higher likelihood of developing new-onset AF.

In contrast, the majority of strokes after non-cardiac surgery were ascribed to cerebrovascular thrombosis rather than embolism [33]. Inflammation has been proposed as a major culprit in the pathogenesis and progression of atherosclerosis and thrombosis [34]. The systemic inflammatory response triggered by surgery, as evidenced by the increase of various cytokines [35], may precipitate cerebrovascular thrombosis. Besides, endothelial dysfunction after non-cardiac surgery may also contribute to the risk of stroke [36]. Endothelial dysfunction in vessels may cause reactive vasospasm and plaque rupture, leading to formation of thrombus with acute ischemia [33]. Furthermore, failure to resume statins after surgery due to complications (e.g., ileus after digestive surgery) may further augment inflammation and thrombosis [37]. All these mechanisms may underlie the increased risk of stroke in the later phase after surgery.

Notably, musculoskeletal surgery increased the risk of stroke within 30 days in the case-crossover analysis, but its effect on stroke became neutral in the case-time-control analysis. In these analyses, we adjusted for several factors including the mode of anesthesia, a potentially modifiable risk factor for orthopedic joint replacement [4]. Because the study population comprised only patients who were discharged free of stroke after surgery, we speculated that intraoperative and immediately postoperative mechanisms (e.g. embolism or hypotension during general anesthesia) may play a dominant role in the development of ischemic stroke after musculoskeletal surgery. A previous study also found that the risk of stroke was highest during the first two weeks following total hip replacement [38].

Some limitations in the present study should be addressed. First, the number of patients who underwent certain types of surgery such as obstetric/gynecologic surgery was small and might be insufficient to detect a statistically significant effect. Second, even though we have controlled for medications including antithrombotic therapy based on prescription claims, we were unable to determine whether and how long medications were withdrawn during the perioperative period, as well as whether medications were actually taken as prescribed. However, a meta-analysis of randomized controlled trials found that perioperative aspirin therapy had no effect on the occurrence of arterial ischemic events [39]. Therefore, we believe that the results may not be affected. Third, surgical types were classified based on the ICD-9-CM procedure codes. Study results based on different classification schemes may not be readily comparable. For example, vascular surgery in this study encompassed various operations on arteries and veins. We were unable to determine whether an operation was related to carotid arteries or not. Fourth, the mechanisms of stroke related to different types of surgery could not be established based on the current dataset. Finally, we have to recognize a shortcoming of this claims-based study, that is, the potential for miscoding.

## Conclusions

Overall, patients who were discharged without stroke from inpatient surgery carried an increased risk of ischemic stroke within 30 days. Patients undergoing cardiothoracic and vascular surgery were, not surprisingly, at high risk for ischemic stroke. Among the remaining types of surgery, only digestive surgery independently increased the risk of ischemic stroke. Further studies are warranted to determine the underlying mechanisms as well as to seek appropriate preventive strategies for stroke within 30 days after surgery.

## Supporting information

S1 Fig Flow chart showing the derivation of the study population. ICD-9-CM, International Classification of Diseases, Ninth Revision, Clinical Modification.
(PDF)

S1 Table Surgical types. (PDF)

S2 Table Comorbidities. (PDF)

S3 Table Medication use. (PDF)

S4 Table Risk of ischemic stroke associated with various types of surgery using case-crossover and case-time-control analyses. (PDF)

S5 Table Comorbidities and medication use between the case and control groups in the case-time-control analysis. (PDF)
