# Supplementary figures and images for: Risk of ischemic stroke after discharge from inpatient surgery: Does the type of surgery matter?
Source: PLoS One. 2018 Nov 5;13(11):e0206990. doi: 10.1371/journal.pone.0206990 (PMC6218083; doi:10.1371/journal.pone.0206990)

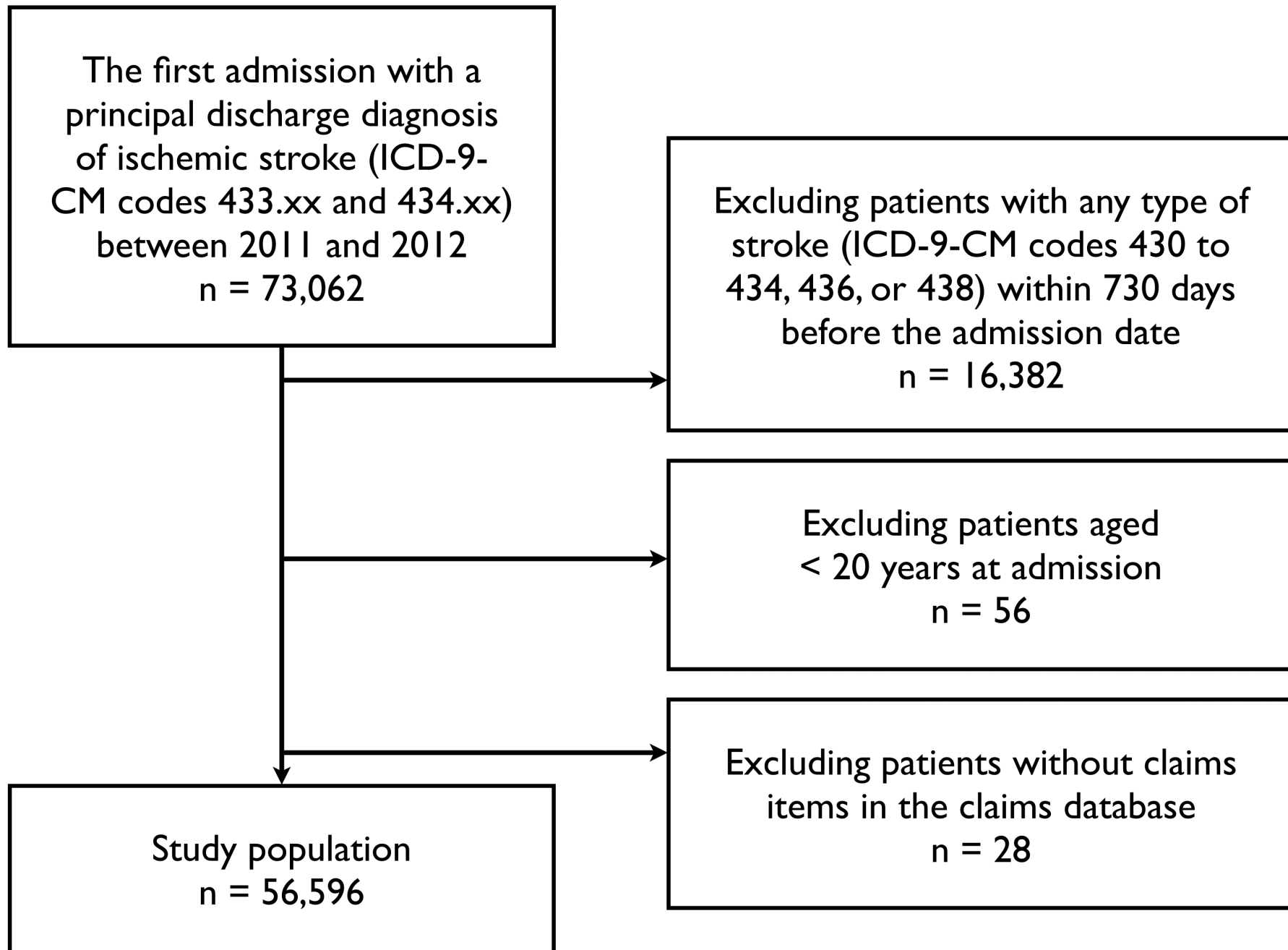

Supplement: S1 Fig — ICD-9-CM, International Classification of Diseases, Ninth Revision, Clinical Modification. (PDF) [file pone.0206990.s001.pdf]
