# Supplementary material for: Risk of ischemic stroke after discharge from inpatient surgery: Does the type of surgery matter?
Source: PLoS One. 2018 Nov 5;13(11):e0206990. doi: 10.1371/journal.pone.0206990 (PMC6218083; doi:10.1371/journal.pone.0206990)
Supplement: S1 Table — (PDF) [file pone.0206990.s002.pdf]

S1 Table. Surgical types.

Subtype	ICD-9-CM procedure codes
**Nervous**	01–05
**Eye**	08–16
**Ear/nose/mouth/pharynx**	18–29
**Cardiothoracic**	30–37
**Vascular**	38–39
**Digestive**	42-54
**Genitourinary**	55–64
**Obstetric/gynecologic**	65–75
**Musculoskeletal**	76–84
**Integumentary**	85–86
**Miscellaneous**	06–07, 40–41

ICD-9-CM, International Classification of Diseases, Ninth Revision, Clinical Modification.
