# Supplementary material for: Risk of ischemic stroke after discharge from inpatient surgery: Does the type of surgery matter?
Source: PLoS One. 2018 Nov 5;13(11):e0206990. doi: 10.1371/journal.pone.0206990 (PMC6218083; doi:10.1371/journal.pone.0206990)
Supplement: S2 Table — (PDF) [file pone.0206990.s003.pdf]

S2 Table. Comorbidities.

Comorbidity	ICD-9-CM diagnosis codes
**Hypertension**	401, 402, 403, 404, 405
**Diabetes mellitus**	250
**Hyperlipidemia**	272.0, 272.1, 272.2, 272.3, 272.4, 272.9
**Atrial fibrillation**	427.31
**Coronary artery disease**	410, 411, 412, 413, 414
**Congestive heart failure**	402.01, 402.11, 402.91, 404.01, 404.03, 404.11, 404.13, 404.91, 404.93, 425, 428
**Chronic kidney disease**	585
**Chronic obstructive pulmonary disease**	491, 492, 493, 494, 496
**Peripheral artery disease**	440, 441, 442, 443, 444, 447, 557
**Transient ischemic attack**	435
**Cancer**	140–208

ICD-9-CM, International Classification of Diseases, Ninth Revision, Clinical Modification.
