# Supplementary material for: Risk of ischemic stroke after discharge from inpatient surgery: Does the type of surgery matter?
Source: PLoS One. 2018 Nov 5;13(11):e0206990. doi: 10.1371/journal.pone.0206990 (PMC6218083; doi:10.1371/journal.pone.0206990)
Supplement: S3 Table — (PDF) [file pone.0206990.s004.pdf]

S3 Table. Medication use.

Medications	ATC codes
**ACE inhibitors or ARBs**	C09
**Beta blockers**	C07
**Calcium channel blockers**	C08
**Diuretics**	C03
**Other antihypertensives**	C02
**Oral antidiabetic drugs**	A10B
**Insulins**	A10A
**Lipid lowering agents**	C10
**Antiplatelets**	B01AC
**Oral anticoagulants**	B01AA, B01AE, B01AF
**NSAIDs**	M01A (excluding M01AX05), M01B
**Antipsychotics**	N05A

ACE, angiotensin-converting enzyme; ARB, angiotensin receptor blocker; ATC, Anatomical Therapeutic Chemical; NSAID, nonsteroidal anti-inflammatory drug.
