# Supplementary material for: Risk of ischemic stroke after discharge from inpatient surgery: Does the type of surgery matter?
Source: PLoS One. 2018 Nov 5;13(11):e0206990. doi: 10.1371/journal.pone.0206990 (PMC6218083; doi:10.1371/journal.pone.0206990)
Supplement: S4 Table — (PDF) [file pone.0206990.s005.pdf]

S4 Table. Risk of ischemic stroke associated with various types of surgery using case-crossover and case-time-control analyses.

	Case-crossover OR (95% CI)[a]			Case-time-control OR (95% CI)[b]		
**Surgery**	**1–30 d**	**31–60 d**	**61–90 d**	**1–30 d**	**31–60 d**	**61–90 d**
**Nervous**	1.05 (0.48–2.31)	1.15 (0.40–3.31)	3.99 (0.85–18.79)	0.38 (0.08–1.81)	0.72 (0.17–3.11)	3.21 (0.33–31.06)
**Eye**	1.61 (0.74–3.49)	0.71 (0.32–1.55)	1.31 (0.50–3.47)	0.73 (0.24–2.25)	0.49 (0.17–1.45)	3.35 (0.93–12.00)
**Ear/nose/ mouth/ pharynx**	0.66 (0.32–1.35)	1.52 (0.58–3.97)	0.90 (0.40–2.00)	0.86 (0.27–2.74)	3.79 (0.99–14.56)	0.69 (0.15–3.10)
**Cardiothoracic**	1.89 (1.41–2.54)	1.25 (0.91–1.73)	0.97 (0.71–1.33)	3.20 (1.89–5.41)	1.92 (1.14–3.22)	1.29 (0.78–2.14)
**Vascular**	1.59 (1.01–2.50)	1.49 (0.94–2.37)	1.26 (0.79–2.01)	2.21 (1.02–4.81)	0.95 (0.35–2.56)	1.18 (0.48–2.87)
**Digestive**	1.76 (1.40–2.21)	1.11 (0.88–1.41)	0.85 (0.66–1.08)	1.64 (1.15–2.34)	1.04 (0.73–1.50)	0.84 (0.58–1.22)
**Genito-urinary**	1.21 (0.82–1.78)	1.32 (0.81–2.14)	1.00 (0.62–1.59)	1.16 (0.63–2.11)	1.63 (0.85–3.13)	0.93 (0.49–1.75)
**Obstetric/ gynecologic**	2.10 (0.69–6.46)	0.93 (0.34–2.59)	0.74 (0.21–2.60)	2.98 (0.57–15.54)	0.78 (0.19–3.19)	3.31 (0.47–23.17)
**Musculoskeletal**	1.30 (1.02–1.66)	0.87 (0.68–1.12)	0.89 (0.68–1.16)	1.07 (0.75–1.52)	0.83 (0.57–1.20)	0.98 (0.67–1.44)
**Integumentary**	1.27 (0.81–2.00)	1.41 (0.78–2.55)	1.00 (0.58–1.72)	0.60 (0.26–1.42)	0.82 (0.32–2.13)	1.53 (0.62–3.76)
**Miscellaneous**	2.40 (0.75–7.70)	0.58 (0.19–1.79)	0.21 (0.02–2.35)	1.50 (0.24–9.52)	0.33 (0.06–1.95)	0.29 (0.02–4.35)

OR, odds ratio; CI, confidence interval.

[a]Adjusted for health care utilization, comorbidities, discordant use of medications, and mode of anesthesia.

[b]Obtained from the interaction term between surgery and group (case with ischemic stroke versus control without ischemic stroke) and adjusted for health care utilization, comorbidities, discordant use of medications, and mode of anesthesia.
