# Supplementary material for: Risk of ischemic stroke after discharge from inpatient surgery: Does the type of surgery matter?
Source: PLoS One. 2018 Nov 5;13(11):e0206990. doi: 10.1371/journal.pone.0206990 (PMC6218083; doi:10.1371/journal.pone.0206990)
Supplement: S5 Table — (PDF) [file pone.0206990.s006.pdf]

S5 Table. Comorbidities and medication use between the case and control groups in the case-time-control analysis.

	Control (n = 54384)	Case (n = 54384)	*P*
**Comorbidity, %**			
**Hypertension**	42.6	54.8	<0.001
**Diabetes mellitus**	18.4	31.0	<0.001
**Hyperlipidemia**	17.8	19.4	<0.001
**Atrial fibrillation**	1.6	4.9	<0.001
**Coronary artery disease**	12.6	16.0	<0.001
**Congestive heart failure**	4.6	8.7	<0.001
**Chronic kidney disease**	2.9	4.9	<0.001
**Chronic obstructive pulmonary disease**	8.9	9.2	0.034
**Peripheral artery disease**	2.2	3.3	<0.001
**Transient ischemic attack**	0	2.2	<0.001
**Cancer**	5.6	5.4	0.155
**Health care utilization, median (IQR)**	0 (0–0)	2 (0–4)	<0.001
**Medication, %**			
**ACE inhibitors or ARBs**	24.9	33.9	<0.001
**Beta blockers**	16.9	24.5	<0.001
**Calcium channel blockers**	26.1	33.8	<0.001
**Diuretics**	10.2	15.9	<0.001
**Other antihypertensives**	4.9	6.1	<0.001
**Oral antidiabetic drugs**	15.5	25.7	<0.001
**Insulins**	1.8	5.5	<0.001
**Lipid lowering agents**	13.4	15.8	<0.001

**Antiplatelets**	17.7	26.8	<0.001
**Oral anticoagulants**	0.8	2.0	<0.001
**NSAIDs**	36.3	39.0	<0.001
**Antipsychotics**	3.7	6.6	<0.001

ACE, angiotensin-converting enzyme; ARB, angiotensin receptor blocker; IQR, interquartile range; NSAID, nonsteroidal anti-inflammatory drug.
